# Advanced Maternal Age in IVF: Still a Challenge? The Present and the Future of Its Treatment

**DOI:** 10.3389/fendo.2019.00094

**Published:** 2019-02-20

**Authors:** Filippo Maria Ubaldi, Danilo Cimadomo, Alberto Vaiarelli, Gemma Fabozzi, Roberta Venturella, Roberta Maggiulli, Rossella Mazzilli, Susanna Ferrero, Antonio Palagiano, Laura Rienzi

**Affiliations:** ^1^Clinica Valle Giulia, G.en.e.r.a. Centers for Reproductive Medicine, Rome, Italy; ^2^Department of Experimental and Clinical Medicine, University Magna Græcia of Catanzaro, Catanzaro, Italy; ^3^Andrology Unit, Department of Clinical and Molecular Medicine, Sant'Andrea Hospital, Sapienza University of Rome, Rome, Italy; ^4^Department of Gynecological, Obstetrical and Reproductive Sciences, University of Campania Luigi Vanvitelli, Caserta, Italy

**Keywords:** advanced maternal age, ovarian stimulation, embryo selection, single embryo transfer, oocyte donation, oocyte cryopreservation

## Abstract

Advanced maternal age (AMA; >35 year) is associated with a decline in both ovarian reserve and oocyte competence. At present, no remedies are available to counteract the aging-related fertility decay, however different therapeutic approaches can be offered to women older than 35 year undergoing IVF. This review summarizes the main current strategies proposed for the treatment of AMA: (i) oocyte cryopreservation to conduct fertility preservation for medical reasons or “social freezing” for non-medical reasons, (ii) personalized controlled ovarian stimulation to maximize the exploitation of the ovarian reserve in each patient, (iii) enhancement of embryo selection via blastocyst-stage preimplantation genetic testing for aneuploidies and frozen single embryo transfer, or (iv) oocyte donation in case of minimal/null residual chance of pregnancy. Future strategies and tools are in the pipeline that might minimize the risks of AMA through non-invasive approaches for embryo selection (e.g., molecular analyses of leftover products of IVF, such as spent culture media). These are yet challenging but potentially ground-breaking perspectives promising a lower clinical workload with a higher cost-effectiveness. We also reviewed emerging experimental therapeutic approaches to attempt at restoring maternal reproductive potential, e.g., spindle-chromosomal complex, pronuclear or mitochondrial transfer, and chromosome therapy. *In vitro* generation of gametes is also an intriguing challenge for the future. Lastly, since infertility is a social issue, social campaigns, and education among future generations are desirable to promote the awareness of the impact of age and lifestyle habits upon fertility. This should be a duty of the clinical operators in this field.

## Introduction

Advanced maternal age (AMA) is a critical social and clinical issue. Currently, the proportion of women delaying childbearing until the late 3rd–early 4th decade of life has greatly increased, especially in Western societies (1, 2). The reasons can be associated with increased education and woman employment, career goals, highly-effective contraceptive strategies, paucity of social incentives to support parenthood, as well as a diffused and misleading idea that IVF can compensate for the natural decline in infertility with aging (1, 3). The misperception about the resolutive effect of IVF is due to both a lack of knowledge and its growing popularity. This trend is challenging for fertility specialists who are witnessing an increase in the number of women seeking a pregnancy who are older than 35 year, namely the cut-off age to consider a patient of AMA (4). Such threshold is mainly based upon a genetic background: women older than 35 year experience a dramatic increase in embryo aneuploidy rate from a 30% baseline production up to 90% in their late 40s prior to the menopause (5, 6). Specifically, the chance of producing a chromosomally-normal blastocyst might be even lower than 5% in women older than 43 year (7, 8). This can be attributed, on the one hand to the gradual depletion of the ovarian reserve, and on the other hand to the progressive decrease in oocyte/embryo competence, defined as the ability to produce a live birth (9–11). A number of processes have been suggested as causative for the latter: dysfunctional cohesins (12), reduced stringency of spindle-assembly checkpoint (SAC) (13–15), shortening of telomeres (16, 17), and impaired mitochondrial metabolic activity (18, 19). All these processes are directly or indirectly involved in proper chromosome segregation, and therefore in modulating embryo competence (11).

In AMA patients, an infertility work-up is recommended already after 6 months of regular unprotected intercourse, since the impact of time upon couple's reproductive chances clearly exceeds any other putative cause of infertility (20–22). A multi-marker approach to evaluate the ovarian reserve has been proposed, which mainly encompasses basal follicle stimulating hormone (FSH), anti-mullerian hormone (AMH) and antral follicle count (AFC) (23). Moreover, many other factors might impact woman reproductive potential, therefore to rule them out some other investigations are recommended, such as thyroid function, coagulation disorders, previous chlamydial infections, tubal patency, and sperm quality. A thorough counseling is also pivotal, which must cover any possible gestational complication, such as hypertension, diabetes, preeclampsia, placental abruption, intrauterine growth restriction, placenta previa, low birth-weight, pre-term delivery, fetal deaths, and a higher incidence of obstetrical conditions: a glance upon pre-conception education is the very first step for a physician to counteract misinformation (20).

**Figure 1:**
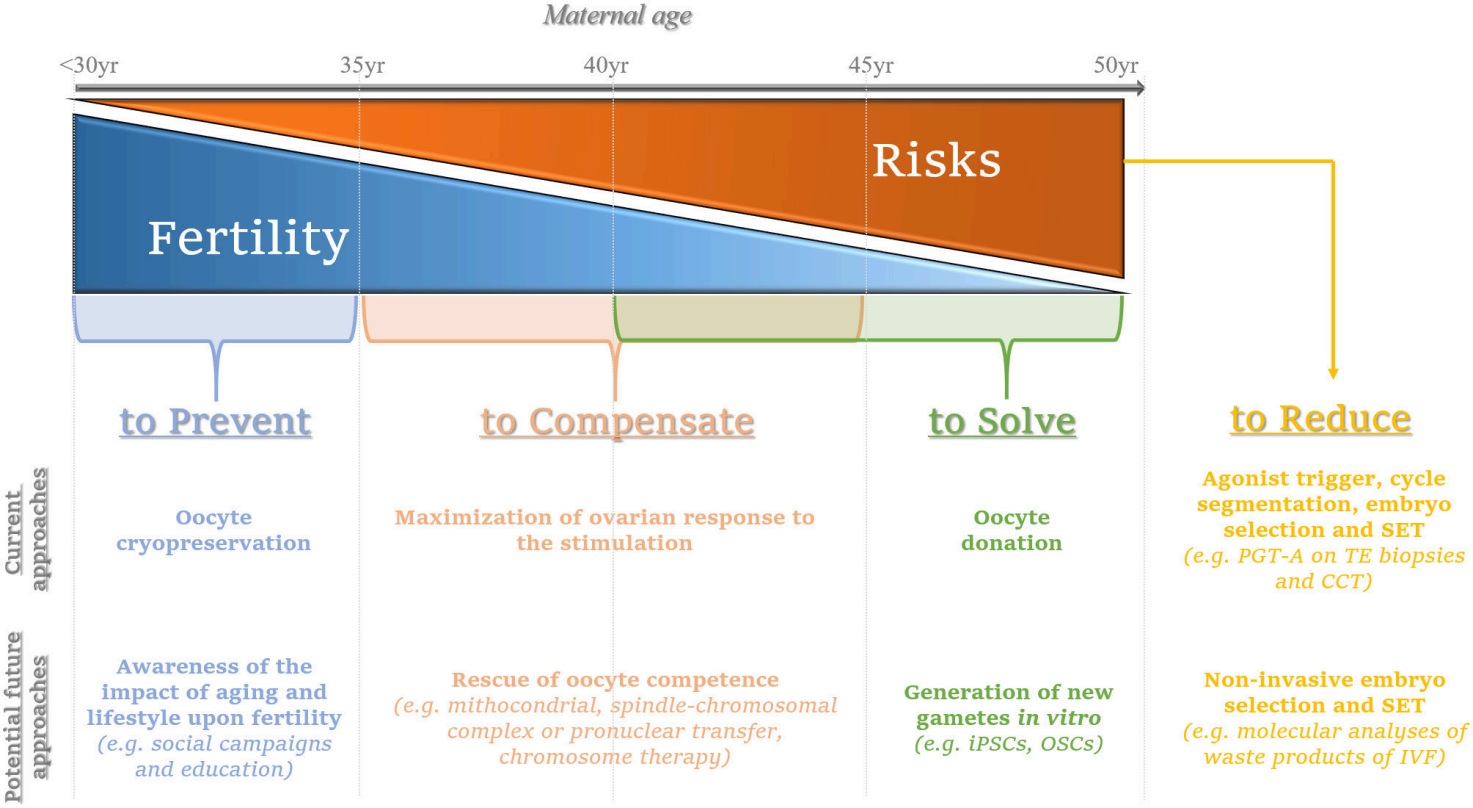
Summary of the current and potential future approaches to prevent, compensate or solve the issues related with advanced maternal age on infertility, while limiting the putative concurrent risks. The consequences deriving from the onset of aging-related female infertility might be prevented by performing oocyte cryopreservation ideally when women are younger than 35 year; in the future, social campaigns and education are advisable to promote the awareness of the age-related female fertility decay. The main current strategy to compensate for the consequences of aging on oocyte competence (woman age range: 35–45 year) entails the maximization of the ovarian response by tailoring patients-specific protocols; in the future, oocyte competence might be rescued via promising approaches such as mitochondrial, spindle-chromosomal complex, pronuclear transfer or chromosome therapy, even though all these perspectives still need thorough and careful investigation. Oocyte donation represents the main current option to solve irrecoverable aging-related infertility conditions (woman age range: 40–50 year); the future avant-gardes that are motivating the academic research instead entail the generation of new gametes *in vitro* [e.g., from induced pluripotent stem cells (iPSCs) or oogonial stem cells (OSCs)]: an intriguing challenge with yet unpredictable outcomes. At last, the increasing maternal age brings about greater reproductive risks for the woman, the pregnancy she will eventually achieve and the new-born: to date, embryo selection via preimplantation genetic testing of aneuploidies (PGT-A) performed by molecular platforms for comprehensive chromosome testing (CCT) on trophectoderm (TE) biopsies represents the most efficient workflow to counteract the hazards derived from embryo chromosomal aneuploidies. Agonist trigger and cycle segmentation are important to minimize the risk of ovarian hyperstimulation syndrome (OHSS) after IVF. Moreover, to avoid the onset of multiple gestations, single embryo transfer (SET) is strongly recommended, especially for euploid blastocysts. In the future, molecular analyses (DNA, mRNA, miRNA, and/or proteins) on leftover products of IVF retrieved via non-invasive procedures (e.g. cumulus cells, spent culture media after IVF) might be implemented clinically to complement (or even replace) invasive approaches of embryo selection thereby aiming at a successful SET. From left to right, the gray arrow represents the increasing maternal age from <30 to 50 year The blue triangle depicts the decreasing maternal fertility, while the orange triangle depicts the increasing risks that could derive from the treatment of infertility and/or the establishment of a pregnancy as the maternal age increases. In light blue, the current and emerging approaches to prevent the fertility decay. In light orange, the current and emerging approaches to compensate it. In green, egg donation is proposed as a current strategy to circumvent the aging issue of female gametes, while emerging still experimental approaches aim at solving this issue. In yellow, we summarized the current strategies recommended to limit the putative risks of an IVF treatment; in the future, non-invasive approaches for embryo selection might be implemented in this workflow to identify the embryo for SET.

The aim of this review is to focus on current and emerging experimental therapeutic approaches for AMA patients undergoing IVF (for a summary see Figure 1).

## Current Management of AMA Patients in IVF

Even if infertility is classified as a disease by the World Health Organization (WHO) (24), it does not automatically require a medical treatment. Therefore, the physicians must propose a treatment based on scientific evidences as well as patient-specific careful investigation: any therapeutic approach must be planned together with the couple attempting to achieve the best clinical outcomes with respect to patients' will and possibilities. In this scenario, the counseling is critical, especially in AMA patients.

The management of AMA should be based on female age, a correct evaluation of the ovarian reserve and additional factors that can impair each couple's chance to conceive. The well-known opposite trend between the increasing aneuploidy rate and decreasing ovarian reserve with maternal aging outlines a thorny situation: in older patients producing less oocytes, far more eggs are required than in young patients to identify at least one chromosomally-normal (i.e., euploid) embryo during an IVF cycle. Recently, we estimated that in women aged 35–37, 38–40, 41–42, and >42 year we would need to collect ~5, 7, 10, and 20 oocytes, respectively, to find at least one euploid embryo (8). In other terms, a proper estimation of the ovarian reserve and of the ovarian response to the stimulation complements woman age to achieve a reliable definition of the specific chance to conceive. AMH, AFC, and FSH should be accounted as the most predictive parameters defined to this end (23, 25). Currently in IVF, no therapy exists to restore the intrinsic gamete/embryo competence, hence the clinician can only tailor the proper strategy to maximize the ovarian response and retrieve the highest possible number of oocytes. The expertise of the embryological team (i.e., efficiency in conducting oocyte and embryo manipulation) and a safe *in vitro* culture environment are then essential to safeguard oocyte/embryo competence (26). At last, reliable and informative strategies for embryo selection are pivotal to estimate that competence (27), aiming at an increased IVF efficiency (i.e., higher implantation rate per transfer, lower miscarriage, and possibly shorter time to achieve a pregnancy) and at adopting a single embryo transfer (SET) policy, in turn also minimizing the risk for multiple gestations.

### Maximization of the Ovarian Response

**Figure 2:**
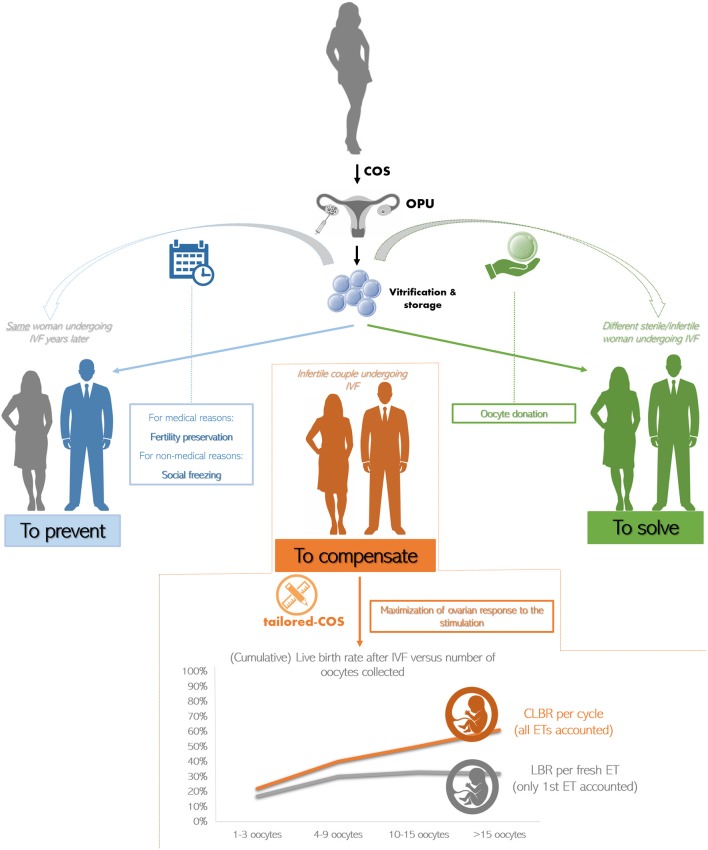
The current clinical strategies to treat advanced maternal age (AMA) in IVF. The oocytes retrieved from a young patient undergoing controlled ovarian stimulation (COS) and oocyte pick-up (OPU) are cryopreserved (i) to be used from the same woman years later thereby preventing the onset of infertility due to medical or non-medical reasons (in blue), or (ii) to be used from a different woman indicated to egg donation (in green). The main strategy to compensate for the age-related infertility in AMA patients is to tailor COS on each woman peculiar characteristics attempting at maximizing the ovarian response. Indeed, while the live birth rate (LBR) per fresh embryo transfer (ET; i.e., only first transfer accounted) plateaus when more than 15 oocytes are retrieved (in gray), the cumulative LBR (CLBR) per cycle (i.e., all consecutive fresh and frozen ETs accounted) instead keeps increasing at any age (in orange). Data adapted from (30).

An accurate estimation of the ovarian reserve, the optimization of the ovarian response and the collection of a consistent number of oocytes represent the ideal workflow to compensate for the reduced competence of the female gametes in AMA patients. Clearly, the main measure of success in any IVF treatment should be the cumulative live birth rate (CLBR) per started cycle, namely the total number of newborns from all the fresh and/or consecutive frozen ETs performed by a couple (28). Indeed, while the target number of oocytes to collect has been set as ~15 if only fresh transfers are accounted (29), when the focus is the CLBR, the larger the cohort of oocytes, the better the outcome (30–33) (Figure 2). Still, these considerations arose from studies mostly targeted to young patients with a good ovarian reserve, rather than to AMA women with a poor prognosis. To this regard, ovarian stimulation can only support the growth of the follicles available during each ovarian cycle, but it cannot generate follicles *ex-novo*. In other terms, it is worthless increasing the dose of gonadotrophins beyond a maximal threshold, which has been set as 300–375 IU/day of FSH plus 75–150 IU/day of LH (34–37) [for a comprehensive review on how ovarian stimulation is conducted and the different regimens see (38–40)]. LH is not always recommended. However, adding LH might be important to promote steroidogenesis and folliculogenesis in specific populations of patients, like AMA women (41). This is due to the effect of aging, which reduces the production of androgens and brings about a decreased ovarian sensitivity and responsiveness to exogenous FSH (42).

Of note, ovarian hyperstimulation syndrome (OHSS) could be one of the most serious iatrogenic complications when controlled ovarian stimulation (COS) protocols are adopted to fully-exploit the ovarian reserve. OHSS is characterized by cystic enlargements of the ovaries and an extra-vascular fluid shift caused by an increased capillarity permeability and by ovarian neo-angiogenesis. Even though OHSS is more frequent in young patients, this condition cannot be excluded in AMA women with a good ovarian reserve. To drastically limit the prevalence of OHSS after IVF, the most recommended strategy is known as “cycle segmentation” (43). According this approach, COS is conducted in a menstrual cycle and the ET of cryopreserved embryos is performed in a following non-stimulated cycle on a physiological endometrium. Usually, the COS protocol used to this end entails gonadotrophins releasing hormone (GnRH) antagonist protocol in combination with gonadotrophins and the use of GnRH agonist to trigger ovulation. Clearly, the systematic cryopreservation of all the oocytes retrieved and/or embryos produced after IVF is mandatory to implement cycle segmentation in an IVF unit.

Across the years, *mild* ovarian stimulation protocols adopting low doses of gonadotrophins have been also proposed to manage infertility in women older than 35 year (44–46). The rationale was to prevent a putative reduction in oocyte and embryo quality that was claimed for convention stimulation protocols. However, some concerns about this *mild* stimulation strategy have been raised: (i) the number of oocytes retrieved and available for fertilization is limited, (ii) more hormonal stimulation cycles and oocyte retrievals might be required to achieve a pregnancy (i.e., longer the time-to-pregnancy), (iii) its cost-effectiveness is still indeterminate, (iv) increased cycle cancellation rate due to no/limited ovarian response derived from its application (47–49). To overcome these important issues, the number of oocytes retrieved must be maximized by fully exploiting each patient's ovarian reserve. This is especially important in AMA women subject to high blastocyst aneuploidy rate (6). The concept of tailoring ovarian stimulation protocols was therefore introduced. A proper dose of gonadotrophins should be outlined to retrieve an ideal number of oocytes and produce a higher number of blastocysts according to patient-specific prognostic features.

As stated previously, a direct correlation exists between the sequential number of oocytes collected and an increased CLBR per started IVF treatment (30–33). Still, when adopting *mild* stimulation approaches, some groups reported that embryos of a higher quality were obtained and that better clinical outcomes were achieved, but only on a per fresh ET perspective (50–52). However, embryos' morphological quality just moderately associates with their chromosomal and reproductive competence (53), and a study design accounting only fresh ETs suffers from at least two limitations. Specifically, a comprehensive clinical picture is missed if accounting only fresh ET (28), and the increased hormonal levels after COS might impair endometrial receptivity, in turn biasing an objective evaluation (54). In contrast, no data instead exist to support an impact of ovarian stimulation on oocyte competence. To conclude then, the full exploitation of the ovarian reserve in AMA should be considered so far the most reasonable strategy to counteract the effect of aging on oocyte competence. Nevertheless, this issue is still controversial. Only large randomized controlled trials (RCTs) will clarify whether *mild* stimulation protocols can be considered adequate for the treatment of AMA.

Several pharmacological co-treatments to COS have been proposed throughout the last decade aiming at an improved IVF outcome in patients of a poor prognosis (i.e., AMA and reduced response to the stimulation). Growth hormone (55, 56), dehydroepiandrosterone (57, 58) or testosterone (59, 60) represent some of the molecules suggested. Yet, the data about a putative increase in oocyte quantity and/or quality are not concordant. RCTs are thus desirable to clarify the potential positive effect of these co-treatments and/or which population might benefit from them.

As mentioned previously, a detailed characterization of each patient is one of the most crucial requirements to improve success in modern IVF. In this regard, the most concerning population of patients is represented by women suffering from low ovarian response after conventional COS. Therefore, a panel of experts known as the POSEIDON (Patient-Oriented Strategies Encompassing IndividualizeD Oocyte Number) group, has recently introduced a novel framework to further improve the classification of these women that encompasses also the issue of AMA. The POSEIDON criteria stratify low prognosis patients in four main categories based on the oocyte yield after COS. In detail, patients with sub-optimal response but normal markers of ovarian reserve were clustered in POSEIDON Group 1 (<35 year) and 2 (≥35 year), while patients with sub-optimal response already predicted by markers of low ovarian reserve were clustered in POSEIDON Group 3 (<35 year) and 4 (≥35 year) (8, 61). POSEIDON Group 1 and 2 might benefit from an increased FSH dosage with the addition of LH during COS to overcome their putative ovarian hyposensitivity to gonadotrophins. Instead, no benefit is predicted by this COS strategy for POSEIDON Group 3 and 4. A more promising approach outlined for them entails either oocyte/embryo accumulation and cryopreservation after consecutive egg retrievals (62–64) or double stimulation in the follicular and luteal phase of the same ovarian cycle (i.e., DuoStim) (65–69). The implementation of the latter approach originated from the intriguing evidence that multiple waves of follicle recruitment may arise in the same ovarian cycle [for a comprehensive review see (70)], also in a phase (i.e., the luteal phase) that in physiological conditions is anovulatory. Such findings can revolutionize the theory behind folliculogenesis with a direct impact upon ovarian stimulation and the way we conceive it (71). Interestingly, accumulating evidence are outlining encouraging clinical results after luteal phase stimulation *per se* in both oncological and poor prognosis patients who must quickly undergo an IVF treatment (72–77). To conclude, DuoStim and luteal phase stimulation *per se* represent intriguing protocols that certainly demand further investigation to define their safety and real clinical efficiency.

### Oocyte Cryopreservation for Medical and Non-medical Reasons

IVF might represent an efficient strategy for infertile couples who wish to postpone parenthood. However, each woman (not only oncological patients) should be opportunely informed about the methods available to preserve her fertility. Until the last decade, embryo freezing was considered the main established strategy to conduct fertility preservation, since oocyte cryopreservation had led to disappointing and generally inconsistent results. However, the option of cryopreserving embryos for fertility preservation is not always feasible, because of a multitude of ethical, legal and moral issues. Moreover, one of the main drawbacks of freezing embryos to preserve fertility is the putative restriction of female reproductive autonomy, which will always be related to the male partner involved at the time of cryopreservation (78).

The enormous improvement in oocyte survival rate reported from the introduction of the vitrification approach (i.e., a fast cryopreservation protocol that avoids the formation of ice crystals, opposed to slow-freezing), led to the definition of oocyte cryopreservation as the gold standard to conduct fertility preservation (79). Today, the efficiency of oocyte vitrification has been further boosted (80), thereby encouraging the clinician to propose oocytes vitrification (also known as eggs-banking) for medical reasons (e.g., cancer, endometriosis; i.e., fertility preservation), as well as to prevent the age-related decline in both quantity and quality of the eggs (non-medical reasons; i.e., “social-freezing”) (81–83). The workflow simply entails COS, transvaginal oocytes retrieval, cryopreservation of the mature oocytes, and long-term storage (Figure 2). Accumulating evidence is outlining this workflow, especially if vitrification is conducted, as an efficient approach in terms of survival rate, unaffected oocyte quality (e.g., no increase in embryo aneuploidy rate and no difference in gene expression), as well as clinical and obstetrical outcomes (80, 84–89). Social-freezing in particular might be considered a “reproductive insurance” against age-related infertility (90, 91), since it reduces the incidence of oocyte donation (OD) and the burden of ineffective fertility treatment at older ages. Nonetheless, AMA could result in other complications beyond oocyte incompetence, namely higher risk for ectopic pregnancy, preeclampsia, delivery by cesarean section, pre-term delivery and low birth-weight, which should not be disregarded (20).

Clearly, woman age at the time of oocyte cryopreservation and the number of oocytes stored represent crucial parameters affecting the cost-effectiveness of oocyte freezing (92, 93). As expected, the efficacy of such procedure in terms of CLBR is higher in women aged <35 year compared to older patients (93, 94). Doyle et al. defined 37 year as the upper age limit to perform oocyte cryopreservation so that the costs and the effectiveness are sufficiently balanced (92). Therefore, a proper counseling to women wishing to perform oocyte cryopreservation is due to make them aware of its efficacy and prevent unrealistic hopes.

Lastly, although still experimental, alternative strategies to eggs-banking for fertility preservation have been proposed. For instance, the cryopreservation of the ovarian tissue or of either immature or *in vitro-*matured oocytes can be attempted if ovarian stimulation cannot be performed. These procedures are the only available alternatives for prepuberal girls, but are not indicated in AMA patients or in patients with a reduced ovarian reserve (95–98). Therefore, the most appropriate fertility preservation strategy has to be chosen according to the age of the patients, the time available and the type of cancer and related risk for ovarian metastasis (79, 99).

### Oocyte Donation

In patients with a clear depletion of the ovarian reserve, or in presence of recurrent IVF failures, especially after several (euploid) ETs, the only options left are adoption or OD (100). OD gives the chance to an infertile/sterile patient to undergo IVF using a cohort of oocytes previously produced by a young donor and cryopreserved to this end (Figure 2; of note, cryopreservation is not compulsory and the donated oocytes can be used also fresh, straight after retrieval).

In 2014, up to 12% of all IVF cycles in U.S. were performed using donor eggs (www.sartcorsonline.com). The high pregnancy rate achieved from women in their 50s is astonishing (>35%). An evidence indirectly suggesting that in AMA patients the endometrium might be receptive and uterus functional, despite the onset of menopause. The main concerns are the ethical and moral uncertainties related with the transfer of an embryo partially non-self. Undoubtedly, the clinical outcomes after OD in AMA patients encourage the couples toward this procedure rather than aiming to achieve a pregnancy with their own eggs. Still, if the patients are to make a last attempt with their own eggs, these women should be guided toward an informed and conscious decision based on the existing clinical evidence. In this regard, a recent multicenter case series of IVF cycles where preimplantation-genetic-testing-of-aneuploidies (PGT-A; *see next paragraph*) was performed by women older than 44 year outlined that no euploid embryo was produced beyond 46 year. Specifically, even though the LBR when an euploid blastocyst is transferred was as high as ~50%, yet the overall chance to conceive per IVF cycle between 44 and 46 year was as low as ~5% (7).

Once a pregnancy is achieved, though, its follow-up is not always easy to manage. Already back in the 90s, especially pre-eclampsia has been associated with AMA, nulliparity and ovarian failure (101). Moreover, aging *per se* (>40 year) is an independent risk for gestational diabetes, thrombophlebitis (102–104), proteinuria, premature rupture of the membranes, hemorrhage, pre-term birth and low birth-weight, intrauterine growth restriction and abnormal placentation (105–107). Indeed, despite the high success rate of OD, recent evidences from reviews and meta-analyses suggest that pregnancies achieved by this strategy showed a significant increase in the likelihood of several obstetrics and maternal complications with respect to pregnancies achieved through IVF with own eggs (108, 109). Interestingly, all these risks were independent of maternal age, suggesting that their causes might be ascribed to an immunological mechanism, e.g., host vs. graft rejection phenomenon (109). OD-derived embryos are in fact totally allogenic to the mother and may cause the onset of an immunologic reaction in the recipient that could impair the process of placentation. While these aspects are often neglected by IVF specialists, these conditions expose the AMA patient to severe adverse outcomes, including pregnancy loss, and even maternal death, that might be even 2–4-fold higher compared to young women (110). Fertility specialists, as well as general gynecologists, should be aware of these risks and acknowledge them to any woman seeking for a pregnancy in her 40s. Any decision that the couple makes should pass through a thorough clinical and ethical discussion.

### Minimizing the Reproductive Risks of AMA: The Role of Preimplantation Genetic Testing of Aneuploidies

Already back in 2003, Land and Evers (reporting the opinion of an ESHRE Consensus) highlighted that safeguarding woman health and achieving a singleton pregnancy are critical in IVF (111). This is particularly true for AMA patients, whose safety should be a primary concern, since the risk of maternal morbidity and mortality significantly increases in their latest reproductive ages (103). At present, a strategy to comply with these premises requires to avoid the transfer of aneuploid embryos, increase the pregnancy rate per transfer especially when performing SET. Such scheme is needed to allow less burden and complications (i.e., miscarriage and multiple gestation), as well as a shorter time invested to achieve a pregnancy, both crucial aspects especially for AMA patients.

**Figure 3:**
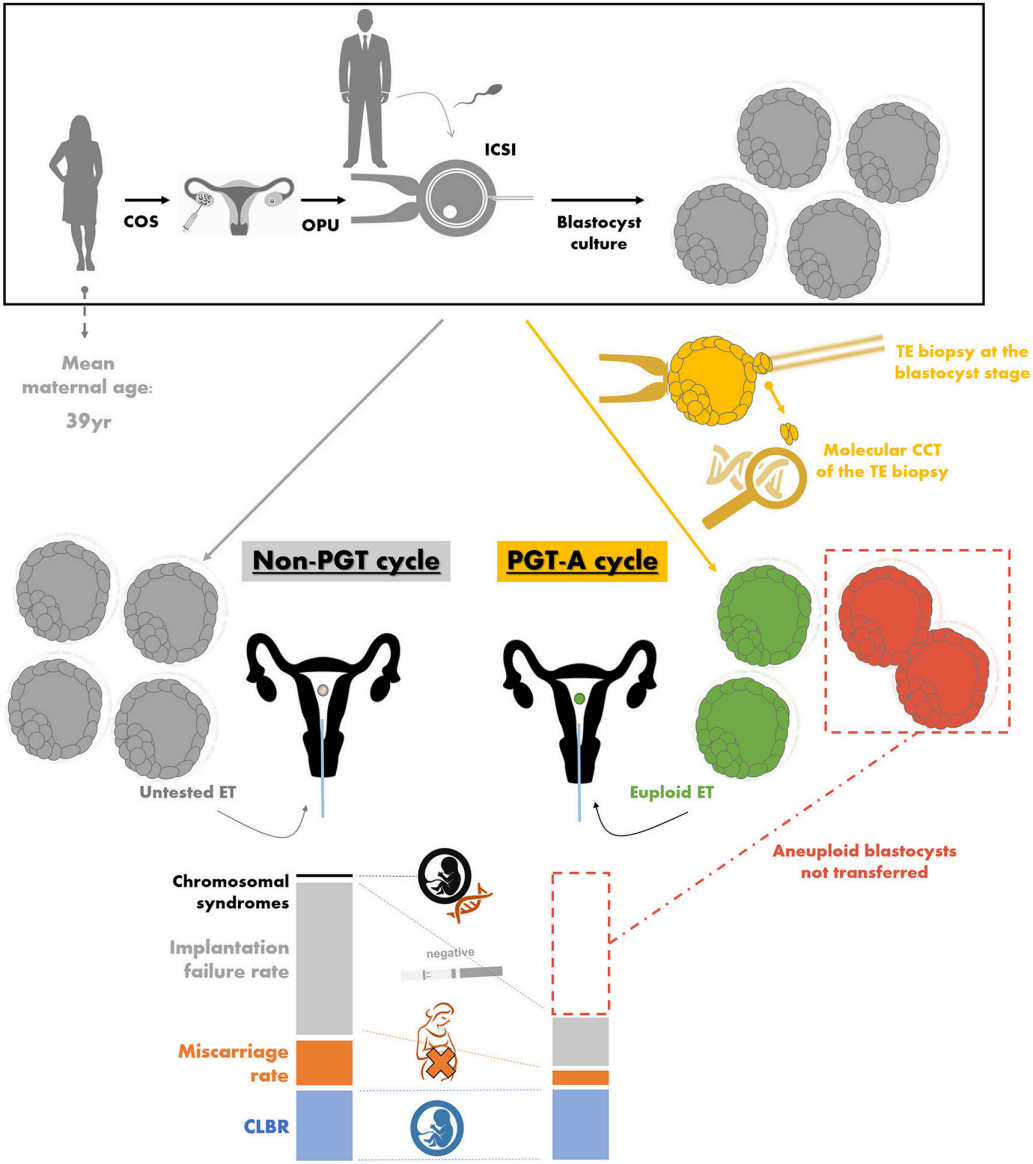
The theory behind preimplantation genetic testing of aneuploidies (PGT-A) and the related workflow. For both non-PGT and PGT-A cycles, a woman undergoes controlled ovarian stimulation (COS), oocyte pick-up (OPU), intracytoplasmatic sperm injection (ICSI) conducted with male partner's sperm, and embryo culture to the blastocyst stage. The differences between non-PGT and PGT-A cycles instead entail for the latter (in yellow): (i) the trophectoderm (TE) biopsy of the blastocysts obtained, (ii) the molecular comprehensive chromosome testing (CCT) of the biopsied fragment, (iii) the definition of euploid blastocysts (in green), which are selected for embryo transfer (ET), and (iv) the definition of aneuploid blastocysts (in red), which are instead prevented from being transferred. In an advanced maternal age (AMA) population (e.g., 39 year mean maternal age), the aneuploidy rate at the blastocyst stage is about 50–55%. In theory, if all the untested blastocysts obtained after a non-PGT and all the euploid blastocysts diagnosed after a PGT-A cycle would be transferred, the latter strategy is expected to bring about (i) the same cumulative live birth rate (CLBR, i.e., the number of babies born per cycle; in blue), (ii) a lower miscarriage rate (in orange), (iii) less ETs resulting in an implantation failure with a negative pregnancy test (in gray), and (iv) no chromosomally-abnormal pregnancy (in black). Data adapted from Capalbo et al. (5), Franasiak et al. (6), Dahdouh et al. (115), Chen et al. (116), and Ubaldi et al. (117).

The only clinical strategy reported to date possibly bringing about all these benefits is PGT-A. This acronym stands for the molecular comprehensive-chromosome-testing (CCT) (i.e., the analysis of the whole karyotype) of an embryo biopsy conducted through quantitative polymerase chain reaction (qPCR), array-comparative genome hybridization (aCGH), single nucleotide polymorphisms-array (SNP-array), or next generation sequencing (NGS) [for a review on these techniques refer to (112)]. Such approach is meant to discriminate euploid from aneuploid embryos in a cohort produced by a couple during an IVF cycle and to transfer only the former, thereby preventing the reproductive risks that might instead derive from the transfer of the latter. Clearly, aneuploidy-testing represents only a tool to conduct an efficient embryo selection and attempt to increase the LBR per set, but it cannot increase the CLBR per cycle (Figure 3). The CLBR is indeed dependent on the intrinsic predisposition of all the embryos obtained after IVF to implant and of the patient to carry a gestation, and aneuploidy testing cannot confer reproductive competence but only provide a tool to estimate such competence. In other terms, in theory, the number of babies born from a cohort of oocytes collected after COS should be the same if aneuploidy-testing is conducted or not, but preventing aneuploid embryos from being transferred should involve a shorter time to reach a pregnancy, a lower risk for miscarriage and a minimal residual risk for vital chromosomal syndromes in the fetus [as low as 0.13% by qPCR and 0.7% by aCGH according to (113, 114)] (Figure 3). To this end, safety and cost-effectiveness are critical parameters, namely embryo competence should not be impacted from the manipulations required for PGT-A and the patients should not be proposed additional procedures in vain.

#### Preimplantation Genetic Testing: The Development of Different Approaches for Embryo Biopsy

Throughout the last 30 years from the first theorization of PGT in the early 90s (118), three settings have been proposed and clinically-adopted: blastomere biopsy at the cleavage stage (i.e., day 3 of embryo preimplantation development), polar bodies (PB) biopsy from the oocytes/zygotes (i.e., day 0–1) and trophectoderm biopsy from the blastocyst (i.e., day 5–7).

- The old-fashioned workflow entailed blastomere biopsy followed by its fluorescent *in situ* hybridization (FISH)-based analysis (i.e., a cytogenetic technique which entails the use of fluorescent probes to selectively bind to specific regions on 9 embryonic chromosomes, thereby outlining a normal or abnormal copy number). However, this approach provided irrelevant benefits and was even detrimental in some cases for AMA patients (119). This outcome was imputed across the years firstly to blastomere biopsy itself, which has been shown to impair embryo implantation potential (120, 121); secondly, to single cell diagnosis, which was not sufficiently-solid and reliable; and lastly, to FISH platform-based analysis, which does not cover the whole embryonic karyotype (i.e., 22 pairs of autosomes and 2 sex chromosomes).
- The setting entailing the biopsy of both PBs from fertilized oocytes is instead time-consuming, since all oocytes should be biopsied regardless their chance to develop to the blastocyst stage. Moreover, it is poorly cost-effective, due to single cells analysis-related limitations and since all PBs should be tested. Finally it may be only partially-informative, since paternal meiotic and mitotic post-zygotic chromosomal missegregations are undetectable on PBs. Yet, PB-based aneuploidy-testing seems not to impair embryo reproductive competence and might be more efficient than conventional IVF. This is what has been reported by the ESHRE Study into the Evaluation of oocyte Euploidy by Microarray analysis (ESTEEM), a recently published RCT conducted in women aged 36–40 year (122). This RCT in fact showed consistent CLBR, but also less transfers, cryopreservation procedures and miscarriages when PBs-based PGT-A was conducted.
- Lastly, blastocyst biopsy setting entails the retrieval of a multicellular fragment (ca. 5–10 cells) from the trophectoderm, which is the section of the embryo that gives origin to the extra-embryonic membranes, while keeping untouched the inner cell mass, which is the section that instead gives origin to the fetus. This approach has been reported safe, standardized, informative, and is implicitly more cost-effective than the previous ones, since only developmentally-competent embryos would reach to the blastocyst stage and be biopsied (112, 120, 121, 123–125). All these evidences involved a wider implementation of trophectoderm biopsy approach so that in 2016 its application has finally outnumbered the other strategies (126).

#### Implementing PGT in the Clinical Management of AMA Patients

At present, cycle segmentation, blastocyst culture, vitrification, blastocyst biopsy with CCT, and SET represent the most promising advances in IVF that, if properly implemented, involve a more efficient and safer treatment (43, 54, 80, 115, 116, 120, 121, 127). Dahdouh and Chen in their two meta-analyses summarizing the RCTs published up to 2015 to investigate PGT-A efficiency, outlined consistently-higher implantation and lower miscarriage rates when euploid blastocysts are transferred (115, 116). Still, most of the evidence were produced in relatively-young patients and the data were reported only from a per transfer perspective. Therefore, the American Society of Assisted Reproductive Technologies (SART) recently recognized the clinical value of blastocyst stage PGT-A, but also requested future investigations to address pending issues. Specifically, “*cost-effectiveness; the role and effect of cryopreservation, time to pregnancy, utility in specific subgroups (such as recurrent loss, prior implantation failure, advanced maternal age, etc.); cumulative success rates over time; and total reproductive potential per intervention*” (128) should be better outlined.

In a retrospective study published in 2015, we highlighted how our clinical efficiency and safety in the treatment of AMA was improved across the years in which we implemented in our practice all the advances listed at the beginning of this paragraph. Specifically a higher application of euploid blastocyst SET resulted in the same CLBR with respect to untested cleavage stage double ET, but also in drastically lower miscarriage and multiple pregnancy rates (117). Likewise, the RCT conducted by Rubio et al. in AMA women, reported a similar efficacy, but a higher efficiency for PGT-A, even if adopting a blastomere biopsy-based CCT approach (129).

A last important advantage of euploid-SET (especially at the blastocyst stage) is that it equals the pregnancy outcomes of double untested blastocyst transfer, but largely limits (virtually abates) multiple pregnancies and their related perinatal and obstetrical complications. An evidence that has been elegantly reported by Forman et al. in their RCT performed in 2013, whose pregnancies were followed-up in a second paper published in 2014 (130, 131).

#### The Relevance of Chromosomal Mosaicism

The main limitation of PGT-A in its current version is represented by chromosomal mosaicism, namely the presence of cells with different karyotypes in the same blastocyst. Errors in chromosomal segregation can in fact occur also during the mitotic divisions post-fertilization. If such errors happen, in the absence of other constitutive meiotic aneuploidies derived from the oocyte and/or the sperm, the deriving blastocyst would be constituted of different euploid and aneuploid populations of cells.

All the papers published to date attempting to define the risk of mosaicism in human blastocysts disaggregated the embryos in the inner cell mass plus 1–3 multicellular sections of the whole trophectoderm, which were then analyzed one at a time. If accounting only whole-chromosome aneuploidies, overall <10% of the disaggregated blastocysts showed results indicative of chromosomal mosaicism (132–138). However, in the clinical setting, only ca. 5–10 trophectoderm cells are analyzed and such small fragment cannot by definition be sufficient to reliably diagnose “mosaicism” in the rest of the embryo (>100 cells). This is known as the intrinsic *sampling bias* and it is an inevitable issue. Therefore, even if healthy pregnancies might be established from the transfer of allegedly-mosaic blastocysts (139–142), still it is difficult to distinguish between false positive calls and genuine mosaicism in the current setting (143–146).

Only positive and negative predictive values (PPV and NPV) generated through a blinded non-selection design could finally provide an accurate definition of the real clinical meaning (and the related risks) of reporting putative mosaicism after PGT-A. In other terms, the trophectoderm biopsy should be retrieved and analyzed, but the blastocysts should be transferred without alleged-mosaicism being disclosed to both the IVF practitioners and the couple. Later on, the results should be finally disclosed to delineate an accurate estimate of the live birth and miscarriage rates deriving from the transfer of allegedly-mosaic blastocysts. Up to date, this design was indeed adopted by Scott et al. back in 2012 (124) to address full aneuploidies (not “mosaic”) detected via SNP-array on trophectoderm-biopsies in a study that still represents a milestone in this field. This study defined a 94% implantation failure and a 48% LBR when “aneuploid” and “euploid” blastocysts were, respectively, transferred in a blinded fashion: those values are still the main clinical first class data currently available in the literature.

The debate upon PGT-A is still open in IVF, therefore specific indications are required and an accurate counseling to the patients is of utmost importance. Still its benefits in AMA patients seem to outnumber the putative limitations, and PGT-A currently represents the only efficient strategy that might limit the age-related reproductive risks in these women. Nonetheless, a successful implementation of PGT-A requires the efficient application of blastocyst culture, biopsy, and vitrification, therefore it must be performed in the hands of experienced IVF units. These prerequisites currently represent important limiting factors to its introduction in the clinical practice worldwide.

## Potential Future Approaches to Treat AMA Infertility

### Minimally- and Non-invasive Embryo Selection

Even if safe and effective, trophectoderm biopsy is a procedure that requires further embryo manipulation, well-experienced and skilled operators, a higher workload for the laboratory, as well as costly instrumentations (e.g., a laser-equipped micromanipulator). Therefore, a quest for non-invasive approaches to conduct embryo selection started in IVF.

Recently, several groups investigated leftover products of IVF for their content in proteins, metabolites and even nucleic acids (e.g., mRNA, miRNA, and DNA). The putative sources encompassed the follicular fluid after egg retrieval, the cumulus cells after oocyte decumulation, the spent media after embryo culture, or the blastocoel fluid in the inner cavity of the embryo after blastulation. However, a future clinical application cannot be envisaged for any of these sources. The molecular approaches to investigate them still need to be refined and tested for their real clinical value before they could be implemented to attempt at improving the cost-effectiveness of CCT (147–152).

The most promising game-changer among the proposed approaches is the screening of genomic-DNA from the spent culture media after IVF (non-invasive PGT). Scrupulous pre-clinical studies are therefore required to validate and improve the contrasting results published to date (153–162). Great efforts have been already made to push the detection limit of CCT down to cell-free DNA; yet, the data are far from being indicative of a short-term clinical implementation (153, 162). IVF clinics and molecular biology laboratories are currently closely collaborating on both the culture and molecular protocols. This multidisciplinary perspective is needed to increase the DNA amplification rate and to characterize the embryonic features and the biological causes related with the presence of cell-free DNA in the culture media. Currently, the main issues are represented by the risk of contamination from exogenous and/or maternal DNA deriving from degenerated PBs or cumulus cells (153, 162). Therefore, future studies should define protocols to avoid or circumvent these limitations.

### Germline Engineering

Even if no clinical therapy is available to counteract the age-dependent fertility decline, recently emerging therapeutic approaches have been proposed to restore the developmental competence of aged oocytes.

**Figure 4:**
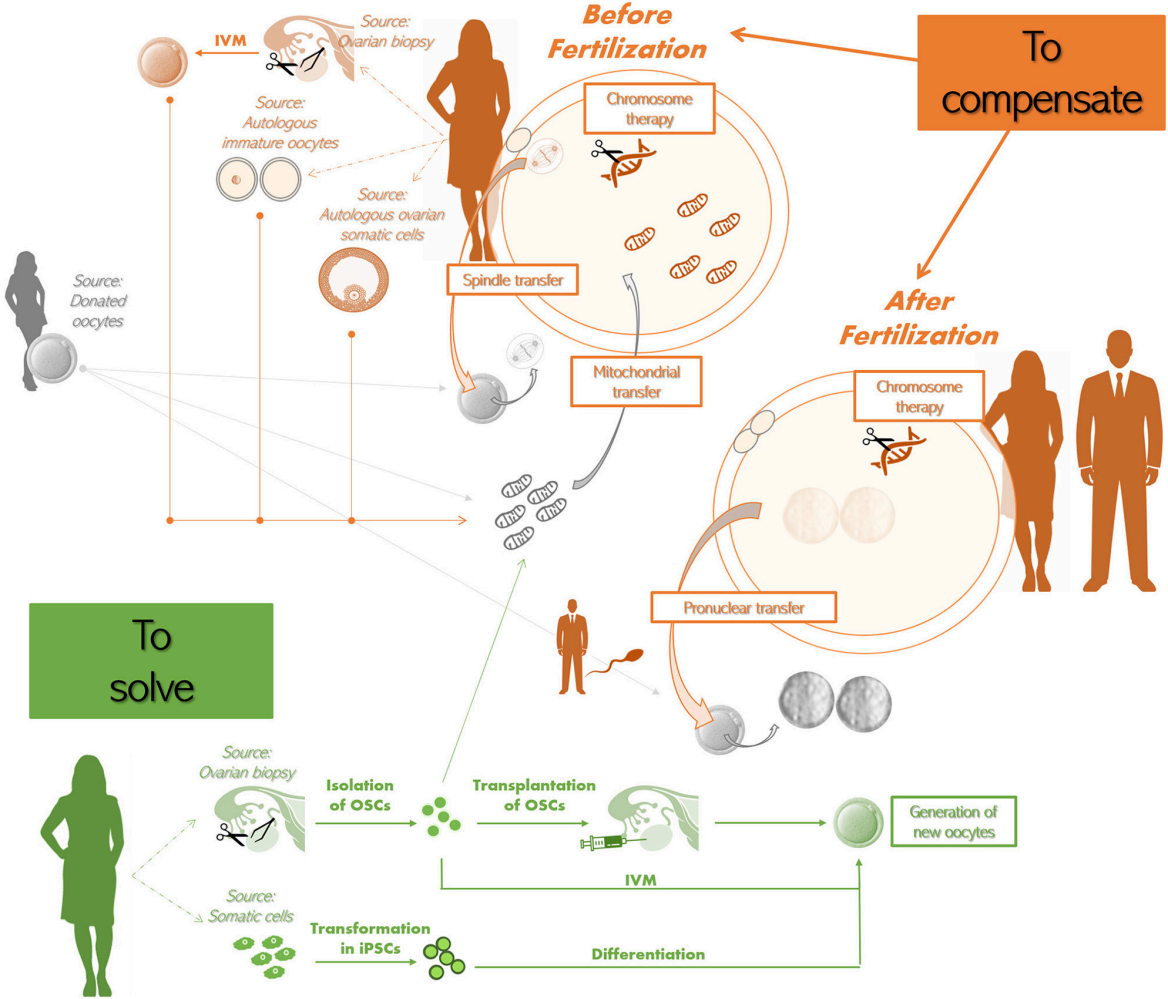
Potential future therapeutic approaches to treat advanced maternal age (AMA) infertility. To compensate before fertilization. The approaches that have been theorized are: •**The spindle-chromosomal complex transfer** from the mature oocyte of an infertile AMA woman (in orange) to the oocyte of a young donor (whose spindle-chromosomal complex was previously removed; in gray). The latter oocyte should be then used to perform IVF; •**The transfer of additional mitochondria** to the oocyte of an infertile AMA woman. The source of the additional mitochondria might be a donated oocyte (in gray). Also autologous sources have been proposed, i.e., oocytes obtained from ovarian biopsies and *in vitro* maturation (IVM), other immature oocytes that cannot be used for IVF, ovarian somatic cells (e.g., cumulus or granulosa cells) (all in orange). Some groups reported that autologous mitochondria can be obtained also from oogonial stem cells (OSCs) isolated from ovarian biopsies (in green); •**Chromosome therapy** to correct meiotic aneuploidies in the oocyte. To compensate after fertilization. The approaches that have been theorized are •The use of the sperm from male partner (in orange) to fertilize both the patient's (in orange) and the donor's egg(s) (in gray), to then remove the pronuclei from the zygote originating from the latter and replace them with the pronuclei from the zygote originating from the former (i.e., **pronuclear transfer**); •**Chromosome therapy** to correct aneuploidies in the zygote. To solve (in green). Two strategies have been theorized: •**The isolation of OSCs** from ovarian biopsies retrieved from the infertile patient to then trigger the formation of new autologous oocytes by either transplanting them back in the ovary or by performing IVM; •The isolation of somatic cells, that are then transformed into **induced pluripotent stem cells (iPSCs)**, which are finally differentiated into new autologous oocytes. Of note, all these putative future therapeutic strategies are still experimental and/or raised biological, genetic, technical, and ethical concerns.

The first strategy proposed entails the replacement of dysfunctional mitochondria, since they represent the main ooplasmic factor determinant for oocyte quality which might be affected by aging (163). The earliest attempt was performed already back in the 90s and entailed the transfer of a small volume of cytoplasm from a presumably-fertile young donor's oocyte into a presumably-defective recipient one. This practice, known as “cytoplasmic transfer,” resulted in about 50 live births (164–166) suggesting that the ooplasm with its components (i.e., mainly mitochondria) might be crucial to confer oocyte competence (Figure 4). Nonetheless, the clinical value of this procedure (in general and to AMA patients) is uncertain and ethical concerns arose also due to unpredictable genetic implications underlying it (e.g., mitochondrial heteroplasmy, namely the presence of two different mitochondrial DNA in the same oocyte). Therefore, the U.S. Food and Drug Administration (FDA) banned it in 2002.

The autologous transfer of mitochondria from the patient's own germ cells allows to circumvent the ethical restriction related to cytoplasmic transfer strategy. Recently, oogonial stem cells (OSCs), i.e., the precursor cells of oocytes, have been proposed as autologous source of germline mitochondria: a further emerging therapeutic approach (167–169). Specifically, autologous additional mitochondria isolated from OSCs of patients' ovarian cortex may be transferred into oocytes by microinjection attempting to restore fertility in poor prognosis women (Figure 4). Additional sources of autologous mitochondria can be represented by somatic cells, but, besides possibly suffering from the same aging-related issues affecting the gamete, the effectiveness of their use might be impacted from tissue-specific characteristics of the mitochondria themselves. Indeed, the most promising results have been obtained using somatic cells of ovarian origin (170) like granulosa or cumulus cells (Figure 4). Other proposed sources of autologous mitochondria are mature oocytes obtained by *in vitro* follicle activation of ovarian cortical biopsies or discarded immature oocytes retrieved after ovarian stimulation (169) (Figure 4).

Although live births have been reported, concerns and skepticism are still in place regarding the efficacy of all these procedures (171). Extensive validation through properly designed large trials employing them in the clinical practice is indeed still strongly needed (172, 173).

A further alternative option to attempt at rescuing oocyte competence is spindle-chromosomal complex transfer, as theorized by Tachibana et al. and practiced in animal models (174), or pronuclear transfer, as previously conducted in developmentally-abnormal embryos (175). Spindle-chromosomal complex transfer practice involves the removal of the spindle from a patient's mature oocyte as well as from a young donor oocyte; the spindle from the former oocyte is then transferred into the ooplasm from the latter (Figure 4). Pronuclear transfer approach instead entails a similar workflow, but in this case the pronuclei are transferred after fertilization has been achieved in both the patient's and donor eggs with the sperm collected from the male partner (Figure 4). However, the safety and efficacy of these approaches are yet to be outlined. Furthermore, in many countries these practices are not allowed and the scientific community raised several ethical concerns. The restored gametes will in fact inherit nuclear genetic material and cytoplasmic components from oocytes retrieved from two different women.

To conclude, all these practices have been described mainly in small proof-of-concept studies and in young patients. Scientific, medical, legal, and ethical implications exist for of all these technologies and need to be elucidated by the competent authority. Only then, “germline engineering” could be considered for any clinical use in humans, either for preventing inheritable diseases such as mtDNA disorders (176), or as an option to attempt at restoring the competence of aged oocytes.

### Chromosome Therapy

Recently, ground-breaking experiments have been performed in both animal models and human cells *ex-vivo* aiming at the definition of molecular strategies to conduct chromosome therapy, namely the correction of aneuploidies in living cells. In the future, such practice might find direct application in the treatment of cancer or chromosomal disorders, but might also apply to the correction of aneuploid germ cells and embryos in IVF.

For instance, in a study *XIST*, which is the non-coding RNA that induces heterochromatinization and inactivation of one of the X chromosomes in the female karyotype, was inserted and transcribed in iPSCs from a Down Syndrome individual. By this mean, Jiang et al. could silence the chromosome 21 in those cells (177). Amano et al. instead corrected trisomy 21 and 18 to euploidy in human cells *ex vivo*. To do so they used Sendai virus vectors and human aneuploid fibroblasts in which they induced the expression of *ZSCAN4*, which in murine embryos ensures genome stability throughout preimplantation development (178). Finally CRISPR/Cas9 system has been used to conduct targeted trisomic chromosomes elimination in murine cultured cells, embryos, and tissues *in vivo*, as well as in human Down Syndrome iPSCs and cancer cells *ex-vivo* (179).

The research in this field has just begun and the efficiency of these approaches, as well as their putative side effects are still unpredictable to date. However, these are fascinating strategies that might find a clinical application in reproductive medicine in the next decades.

### Generation of New Gametes *in vitro*

A very intriguing perspective is certainly the possibility to generate gametes *in vitro* (Figure 4). Murine iPSCs were differentiated into functional oocytes in presence of specific growth-factors by Hayashi et al. for the first time in 2012 (180).

In the last decade, different studies presented important results investigating the existence of OSCs and their capability of creating new oocytes. Tilly and his group (181) were the first authors to question the long-held dogma lasting 50 years according to which no renewable germinal cells are present in the mammalian ovaries postnatally or after irradiation (182). Studying oogenesis in mouse ovaries, the authors reported the presence of mitotically-active OSCs capable of generating new oocytes that can be fertilized to produce viable offspring (Figure 4). Subsequently, several other studies sustained their hypothesis (183–194), whereas others instead criticized their findings supporting that the results were ambiguous and possibly misinterpreted (195–201). Therefore, many questions are still open concerning both their existence in the first place and then their putative clinical usefulness (202). If their existence would be confirmed and their isolation protocol would be reproducible, OSCs might represent an important avant-garde not only for the treatment of AMA.

OSCs might be beneficial for women suffering from primary ovarian insufficiency (203), to restore endocrine function in women suffering from post-menopausal health conditions (204) or for fertility preservation. For this last goal, they should be removed and cryopreserved before chemo-/radio-therapy and then re-implanted soon after. These are just some of their putative clinical uses (183, 203), which are further supported by Zou et al., who claimed that the cryopreservation of OSCs does not jeopardize their proliferative or differentiation capacity (187). Recently, Silvestris et al. demonstrated that OSCs collected from fresh ovarian cortical fragments of non-menopausal and menopausal women are able to differentiate into large haploid oocyte-like cells and enter meiosis under appropriate culture conditions (205). However, the rate of differentiation was low and the results pointed out that the OSCs might be unable to differentiate in menopausal women due to the inactivity of the ovulatory cycle. Furthermore, it cannot be excluded that the oocytes deriving from OSCs isolated from AMA women may be genetically-compromised.

Further research is certainly required in order to provide unequivocal evidence of OSCs existence and, mostly, to understand how could we use them either to improve a woman's ovarian reserve, to treat different disorders or aiming at fertility preservation.

Recently, Herraiz et al. have reported that fertility rescue and ovarian follicle growth can be promoted by bone marrow stem cell infusion in mice. This provides another possibilist alternative to improve follicular development in aged women, or to preserve fertility in oncological and poor responder patients (206).

Clearly, the future might be bright in this field. Great efforts are required, but equal successes could derive. Of note, any novel therapeutic approach must pass through extensive validation before its clinical application, especially concerning its safety (e.g., epigenetic aspects, etc.).

### Education and Prevention

The last but not least important challenge is prevention in the future generations. There is increasing evidence that lifestyle factors such as nutrition, exercise, smoking, and substance abuse can dramatically affect the reproductive competence (207–209). Furthermore, it has been widely shown an alarming lack of knowledge in the young population, including medical students and healthcare professionals, who ignore or underestimate the age-related fertility decline (210, 211). Generating awareness about the impact of aging and lifestyle habits upon fertility in the future generations represents the main social strategy to limit the increasing prevalence of infertility. The issue of infertility and its limitation/prevention should be faced from a multi-disciplinary perspective and embrace also lifestyle. Positive and negative habits are indeed important during the peri-conceptional period (212), and even more during the first 2 years of life of the new-born.

## Author Contributions

DC, AV, GF, RV, and RMag drafted the manuscript. All other authors collaborated in the definition of the topics and their discussion.

### Conflict of Interest Statement

The authors declare that the research was conducted in the absence of any commercial or financial relationships that could be construed as a potential conflict of interest.
